# A Comprehensive View of Microbial Communities in the Laundering Cycle Suggests a Preventive Effect of Soil Bacteria on Malodour Formation

**DOI:** 10.3390/microorganisms10071465

**Published:** 2022-07-20

**Authors:** Marc-Kevin Zinn, Hans-Curt Flemming, Dirk Bockmühl

**Affiliations:** 1 Faculty of Life Sciences, Rhine-Waal University of Applied Sciences, 47533 Kleve, Germany; marc-kevin.zinn@hochschule-rhein-waal.de; 2 Biofilm Centre, University of Duisburg-Essen, Universitätsstrasse 5, 45131 Essen, Germany; hc.flemming@uni-due.de

**Keywords:** washing machine, malodour, next-generation sequencing, *Rhizobium* spp., household

## Abstract

Microorganisms are an important factor in the wash-and-use cycle of textiles since they can cause unwanted aesthetic effects, such as malodour formation, and even pose health risks. In this regard, a comprehensive view of the microbial communities in washing machines and consideration of the microbial contamination of used textiles is needed to understand the formation of malodour and evaluate the infection risk related to laundering. So far, neither the compositions of washing machine biofilms leading to the formation of or protection against malodour have been investigated intensively, nor have microbial communities on used towels been analysed after normal use. Our results link the qualitative and quantitative analysis of microbial communities in washing machines and on used towels with the occurrence of malodour and thus not only allow for a better risk evaluation but also suggest bacterial colonizers of washing machines that might prevent malodour formation. It was shown that soil bacteria such as Rhizobium, Agrobacterium, Bosea, and Microbacterium in particular are found in non-odourous machines, and that Rhizobium species are able to prevent malodour formation in an in vitro model.

## 1. Introduction

Laundering is seen as a role model for circular processes by aiming to restore the original status of textiles as much as possible. Apart from visible dirt, microbial contaminations can be considered one of the most important targets of the laundry action, since insufficiently removed microorganisms pose a health risk or are at least a cause of unpleasantness such as malodour [1,2,3,4,5]. Although the washing machine is an excellent means to remove microbial cells from the textile surface, it also provides good conditions for a variety of microorganisms. Thus, microbial biofilms can be found on different surfaces with water contact inside the washing machine [6,7]. Microbial cells in biofilms are surrounded by a matrix of extracellular polymeric substances (EPSs), determining (amongst other factors) the special properties of biofilm-associated cells, including an increased resilience to external influences, such as biocides or physical impacts. Likewise, biofilms are very stable systems due to their high biodiversity [8,9,10,11].

Biofilms have already been identified and characterized in many domestic areas, e.g., dishwashers [12], shower heads [13], coffee machines [14], food contact surfaces [15], and washing machines [4,6,7]. In the latter, apart the good availability of nutrients [4,7], a regular input of microorganisms via worn clothing, tap water, and air may lead to a complex situation in terms of microbial contamination and colonization of textile and machine surfaces [4,16].

Previous studies identified *Proteobacteria*, *Actinobacteria*, *Firmicutes*, and *Bacteroidetes* as the main bacterial colonizers of washing machines [5,7,17]. More specifically, Nix et al. showed the presence of genera such as *Brevundimonas, Pseudomonas, Rhizobium, Acinetobacter*, and *Ochrobactrum* in the detergent drawer and the rubber sealant [2,7], which was confirmed in 2020 by Jacksch et al., who also found *Moraxella osloensis (M. osloensis)*, a species supposed to be involved in laundry malodour formation [17,18].

As mentioned, apart from the potential infection risk posed by the microbial species present in washing machine biofilms, malodour formation must be considered as one of the major microbiological problems that is perceived by consumers with regards to laundering [2,4], which may have even increased due to the tendency to use lower temperatures in domestic appliances [2,3,4,5]. Malodour is a complex phenomenon that has been described in previous studies. In general, laundry-associated malodour can be divided into two groups: On the one hand, there are sweat-related odours that derive from the microbial metabolization of sweat and/or volatile sweat compounds that are not completely removed during laundering [19,20,21,22]. On the other hand, there are pungent odours that are often described as “wet-and-dustcloth-like”. According to several studies, these odours seem to at least partly be caused by microbial colonizers of the washing machine and might be related to low-temperature laundry [18,23,24]. Although the formation of malodour must be considered a multi-factorial problem, the washing machine as a source of malodorous water-borne and ubiquitous bacteria might play a pivotal role in malodour development [1,25].

There is good evidence that the microbial contamination found on textiles after laundering comprises a mixture of microorganisms originating from the environment or the washing machine and from the human skin [5]. Inter alia, the genera *Staphylococcus*, *Corynebacterium*, and *Micrococcus* are regularly found in this context [2] and were used by Zinn et al. (2021) to simulate the typical “wet-fabric-like” malodour in a laboratory model, where, amongst others, disulfides and indol were identified as marker substances for this type of odour [26]. Interestingly, there are virtually no studies on the amount and type of microorganisms found on textile after normal use. However, this information would be invaluable to estimate the putative risk of contaminated textiles and to define a desirable antimicrobial efficacy delivered by domestic laundering.

To address this issue, the present study aimed to determine the quality and quantity of microbiological contamination in household washing machines and on textiles, resembling an everyday life utilization. Apart from giving a first indication on the possible infection risk by cultural analyses combined with metagenomics data, these data were correlated with the presence of malodour by means of a questionnaire. Thus, this paper attempts to comprehensively describe the hygienic quality of laundry-related surfaces in a normal domestic situation.

## 2. Materials and Methods

### 2.1. Sample Collection

A survey on washing behaviour (see Supplementary Materials Table S1) was conducted in 359 households, 48 of whom made themselves available for further sampling.

Each of these 48 households received a new, comprehensive questionnaire (Table S2) on the number, age, and gender of the members of the household. In addition, the questionnaire asked about pets, illnesses in recent weeks, and visitors during the sampling period. Furthermore, questions were asked about a possible malodour problem, the use of different detergents/additives, and commonly used washing programs.

In each household, the detergent drawer and rubber sealant of the washing machine were sampled using a sterile swab (plain swab sterile plastic applicator rayon-tipped, Copan, Brescia, Italy). For this purpose, an area of 4 cm^2^ was sampled for 1 min and the swab was subsequently transferred to a 2 mL reaction tube filled with 1.5 mL of buffered peptone water (PBS) (Carl Roth GmbH & Co. KG, Karlsruhe, Germany). Samples were stored at 4 °C in the refrigerator until processing.

In addition to the samples from the washing machine, a sterile test towel (Kornan Guest Towel Gray (80% cotton, 20% rayon), IKEA Deutschland GmbH & Co. KG, München, Germany) was given to the households to use for hands in the bathroom for a test period of seven days. During this test phase, the test towel was not allowed to be dried on the heater or washed.

In addition to handing out towels for hands in the bathroom, two other towels (a towel for the body (Kornan guest towel grey (80% cotton, 20% rayon), IKEA Germany GmbH & Co. KG, Germany) and a kitchen towel (Rinnig dish towels (100% cotton), IKEA Germany GmbH & Co. KG, Germany) were given to 20 households. These were also used during the seven-day test phase and were neither washed nor dried over the heater.

### 2.2. Microbial Count on Contaminated Towels

From the used towels, a 4 × 4 cm (16 cm^2^) piece was cut out and placed in a 50 mL falcon tube (Sarstedt AG & Co. KG, Nümbrecht, Germany) filled with 20 mL of sterile 0.9% NaCl. The tubes were placed on a roll shaker (MaxQ*, Thermo Fisher Scientific Inc., Waltham, Massachusetts, USA) for 20 min followed by vigorous vortexing for 1 min and centrifugation for 20 min/8 °C/4696 g.

After centrifugation, the supernatant was discarded and the remaining pellet was resuspended in 700 µL of sterile 0.9% NaCl, which was used both for the determination of the bacterial count and for DNA extraction using the “Fast-DNA SPIN Kit for Soil” (MP Biomedicals GmbH, Eschwege, Germany).

For the determination of the bacterial counts, a decimal dilution series was prepared and 100 µL of each sample was spread on Tryptic Soy Agar to detect aerobic mesophilic bacteria (TSA, Merck KGaA, Darmstadt, Germany), Malt Extract Agar to detect yeasts and moulds (MEA, Merck KGaA, Darmstadt, Germany), MacConkey to detect Gram-negative bacteria (Merck KGaA, Darmstadt, Germany), Mannitol Salt Agar to detect *Staphylococcus* spp. (MSA, Xebios Diagnostics GmbH, Düsseldorf, Germany), and Cetrimide Agar to detect *Pseudomonas* spp. (Xebios Diagnostic GmbH, Düsseldorf, Germany)). Incubation was performed at 30 (MEA, MSA, and Cetrimide) or 37 °C (TSA, MacConkey).

### 2.3. Statistical Methods

After incubation, the exact number of colonies was noted for each plate, but only plates containing between 10 and 300 colonies were counted.

The exact bacterial count was determined using the weighted mean:

$$N=\frac{c}{\left(n_{1}+0.1\times n_{2}\right)d}$$

where *N* is the number of cells per mL in the test suspension;

*c* is the sum of all countable agar plates;
*n*_1_ is the number of cells taken into account in the lower dilution, i.e., 10^−6^;
*n*_2_ is the number of cells taken into account in the higher dilution, i.e., 10^−7^; and
*d* is the dilution factor corresponding to the lower dilution (10^−6^).

The statistics were performed using GraphPad Prism (GraphPad Software Inc., San Diego, CA, USA). Data were expressed as mean values (± standard error). The alpha diversity (Shannon diversity exp (H’)) and Mann–Whitney test (*p* ≤ 0.05) were performed to identify significant differences between the samples. The structure of the microbial community was compared using a principle component analysis (PCA) and a corresponding heatmap using ClustVis [27]. To detect dissimilarities between two samples or environments, the Bray–Curtis dissimilarity was used. The two-way ANOVA was used to determine the significance of the microbial counts.

### 2.4. DNA Extraction and Metagenome Analysis

DNA extraction was performed using “Fast DNA SPIN Kit for Soil” (MP Biomedicals GmbH, Eschwege, Germany). “BIO-Star PCR Master mix (2×) with SybrGreen PCR Master mix blue” was used to amplify the isolated DNA and the PCR reactions were performed in a thermal cycler (Quantstudio 3, Thermo Fisher Scientific, Waltham, MA, USA) (33 cycles, annealing temperature: 55 °C).

Following the PCR, the concentration of the PCR amplificates was determined in each sample. A photometer (BioPhotometer plus, Eppendorf, Hamburg, Germany) was used for the determination. A blank (PCR-grade water) was determined according to the manufacturer’s instructions and the unknown concentrations were measured at an absorbance of 260 nm.

The DNA samples were analysed by an external laboratory (Cegat GmbH, Tübingen, Germany). The shotgun metagenomic sequencing method was used for this purpose. An advantage of this method is that all DNA fragments are sequenced, and thus functional genes related to specific metabolic pathways are also analysed. The amount used for analysis was 0.1 ng and samples were prepared using the Nextera XT kit (Illumnia Inc., San Diego, CA, USA). Assays were performed using the NovaSeq 600 System (Illumnia Inc., San Diego, CA, USA) and a flow cell type of 2 × 100 bp. Samples from the same sampling site, which showed a DNA concentration that was too low, were pooled.

## 3. Results and Discussion

### 3.1. Laundry Routines and Malodour Formation

The data presented in this study were obtained from 359 households, which were asked to answer questions about their laundry routines, used detergents, and, most importantly, their experience with laundry-associated malodour to complement the microbiological analysis, which was subsequently performed in 48 households that agreed to have samples taken.

**Figure 1:**
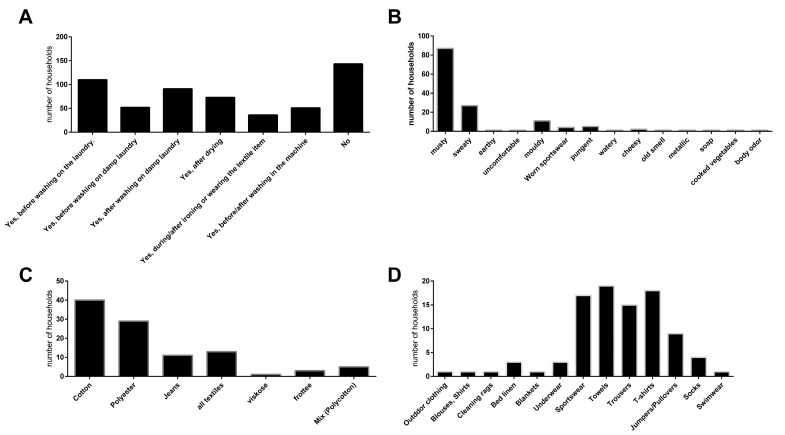
Self-assessment of the surveyed households (*n* = 359) with regards to laundry-related malodour experience (**A**). Households with malodour experience were asked to provide a description of the malodour ((**B**); supported by keywords), the textile mainly associated with malodour (**C**) and the type of garment or textile mainly associated with malodour (**D**).

While the evaluation of the laundry routines, used detergents, and the composition of the households (in terms of children, pets, etc.) did not show any significant correlations with the microbiological parameters, the households could clearly be clustered by their experience with laundry-related malodour (Figure 1).

Of the 359 households, less than half (143 households) reported no experience with malodour. In total, 110 households experienced malodour on laundry before washing while 91 households reported malodour on wet laundry after washing, and 73 households experienced malodour on laundry after drying. The majority of households described the quality of the malodour as musty (87 households). The affected textiles were mainly towels (19 households), T-shirts (18 households), sportswear (17 households), and trousers (15 households) and were predominately made of cotton (40 households) and polyester (29 households).

### 3.2. Microbial Contamination of Washing Machines and Textiles

In the households that agreed to sampling, the detergent drawer and the rubber sealant of the washing machine and the towel used for hands in the bathroom, which was provided and used for seven days, were sampled for an analysis of the microbial communities by plating on selective media and next-generation sequencing after DNA extraction. In addition, in 21 households, a body towel (for use after showering) and a kitchen towel were distributed and analysed accordingly.

**Figure 2:**
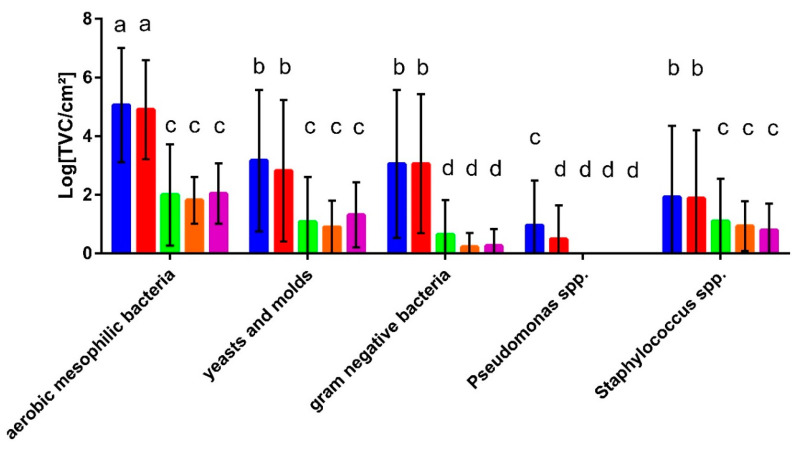
Microbial counts in the detergent drawers (blue) and rubber sealants (red) of the sampled washing machines (*n* = 48) and on the provided and used unwashed hand towels (green; *n* = 48), body towels (orange; *n* = 21), and kitchen cloths (purple; *n* = 21). Different letters indicate significant differences calculated by two-way ANOVA.

The results show that the microbial counts for the total aerobic mesophilic bacteria and all selective media were similar for the detergent drawer and the rubber sealant, except for *Pseudomonas* spp., which showed slightly higher counts in the detergent drawer (Figure 2). The aerobic mesophilic bacterial count for these sampling sites was about 10^5^ cfu/cm^2^ while approximately 10^3^ cfu/cm^2^ of yeasts and moulds and Gram-negative bacteria were present. On the genus level, *Pseudomonas* spp. were present at less than 10 cfu/cm^2^ while approximately 10^2^ cfu/cm^2^ staphylococci were detected. The bacterial counts on the towels and the kitchen cloth were, on average, much lower than those found in the washing machine but, again, were similar for the different sample types. Here, out of the approximately 10^2^ cfu/cm^2^ resembling the total mesophilic count, 1 × 10^1^ cfu/cm^2^ turned out to be fungi or staphylococci, respectively, while Gram-negative bacteria were found to an even lesser extent. In general, no significant differences were found when comparing the microbial counts of households with young (under 35) or old people (over 60), with and without children and households with and without pets.

### 3.3. Metagenome Analysis

**Figure 3:**
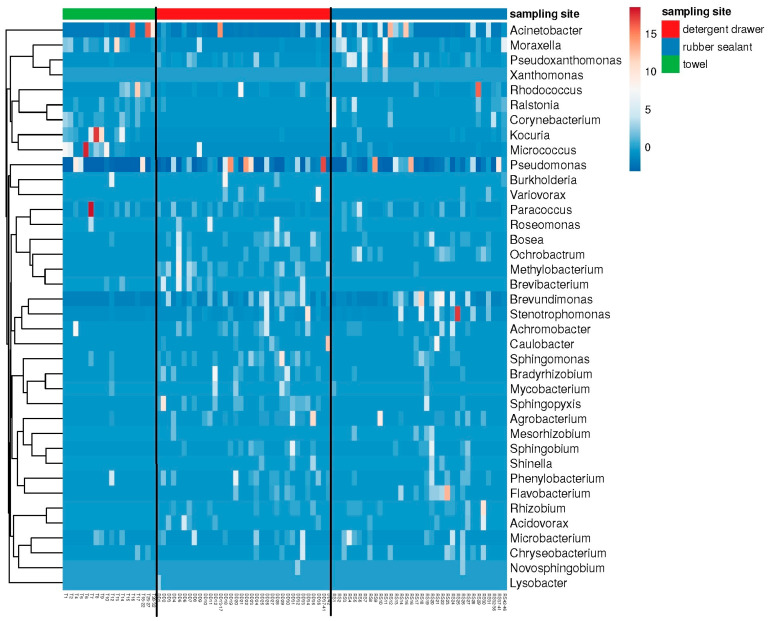
Heatmap showing the main genera present at the different sampling sites. Figure shows “best hits” (i.e., 50% relative frequency) of the metagenome analysis (*n* = 32 towels, 42 detergent drawer, and 46 rubber sealants).

The results of the cultural analysis were partly confirmed and complemented by the metagenome data (Figure 3), revealing the presence of additional genera, with many of them belonging to the family *Pseudomonadaceae* or other Gram-negative bacteria, which are often found in aqueous environments. Moreover, Gram-positive genera, such as *Microbacterium* and *Paracoccus*, were identified, which have previously been described as colonizers of washing machines [2]. Most strikingly, the genus *Rhizobium* was found as well and thus comprises a bacterial genus that can be found in washing machines on a regular basis [2,14]. The most frequently found bacteria, however, belonged to the genera *Pseudomonas* and *Acinetobacter*. The Shannon diversity (not shown) of the bacterial communities was significantly lower (*p* < 0.05) in towels (exp (H’) = 19.97 ± 5.32) and rubber sealants (exp (H’) = 28.96 ± 6.61) compared to the detergent drawer (exp (H’) = 33.06 ± 8.66). In contrast, there were no significant differences in the species richness (towels: *n* = 591 ± 357.9; detergent drawer: *n* = 742 ± 257.1; rubber sealant: *n* = 645 ± 335.6). The beta diversity showed a high degree of variation among the different sampling sites. In particular, the detergent drawer samples were found to have more species in common with the rubber sealant samples (Bray–Curtis index = 0.36) than the towel samples with the detergent drawer (Bray–Curtis index = 0.68) or rubber sealant (Bray–Curtis index = 0.52).

### 3.4. Malodour-Associated Microbial Contamination

**Figure 4:**
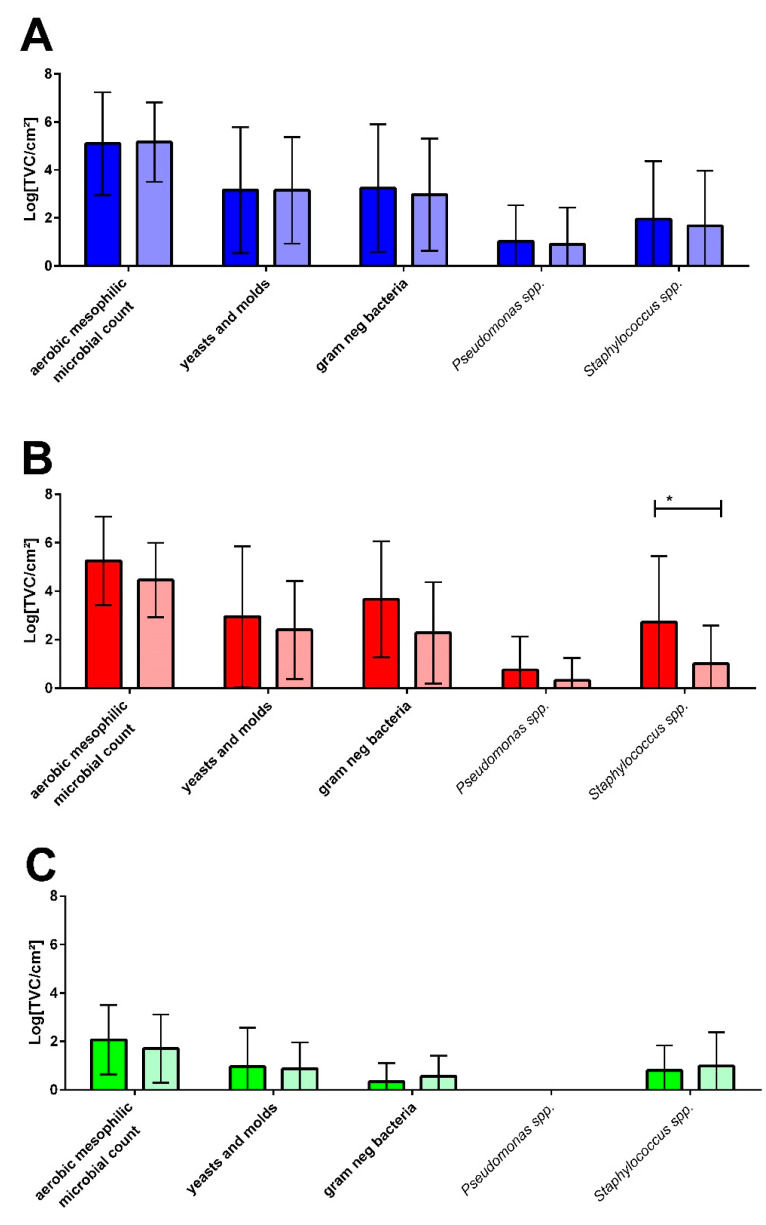
Comparison of the microbial counts in the detergent drawers (**A**), on rubber sealants (**B**), and on used and unwashed towels (**C**) of laundry-related malodour (dark columns, *n* = 16) and non-malodour (light columns, *n* = 26) households. * ≤0.05.

The questionnaire analysis of the 48 households that were sampled revealed 16 households with laundry-related malodour problems, described as “musty” or “mouldy”, while 26 households had no odour problems and 6 households used other descriptive attributes for their laundry odour. Based on this evaluation, the plate count results for the different sampling sites were attributed to the households with malodour (M) or without malodour experience (NM), resulting in the comparative diagram shown in Figure 4.

In general, the microbial counts for all sampling sites and all selective media showed no differences between the M and NM households (Figure 4) except for the rubber sealant, where the mean values tended to be slightly higher and *Staphylococcus* spp. counts were significantly higher in malodour households.

**Figure 5:**
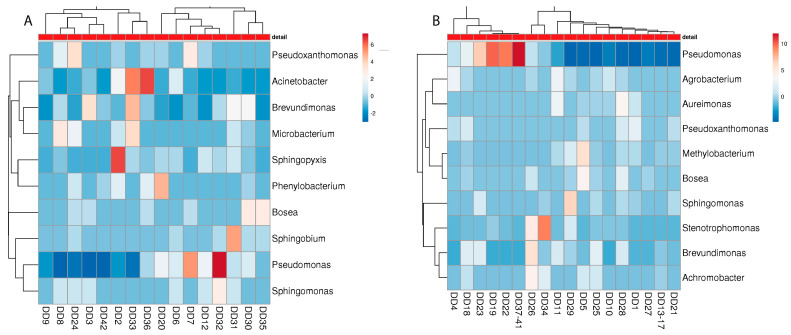
Heatmap of the “best hits” (fractions > 1% of the total quantity) of the bacterial genera identified in the metagenome analysis in different detergent drawers of malodour ((**A**); *n* = 16) and no-odour ((**B**), *n* = 26) households. The number of samples shown may differ from the true sample quantity, as individual samples were pooled in the metagenome analysis.

Considering the presence of distinct genera at the malodour and non-malodour sampling sites, no clear microbial pattern can be associated with either environment. For the detergent drawer, similar species were present in the M and NM machines, with an emphasis on members of *Pseudomonadaceae*, such as *Pseudomonas*, *Pseudoxanthomonas, Sphingomonas*, and *Brevundimonas* (Figure 5). Interestingly, especially in NM machines, various soil bacteria such as *Agrobacterium*, *Bosea*, and *Methylobacterium* were found, out of which only *Bosea* was also present in the M machines.

**Figure 6:**
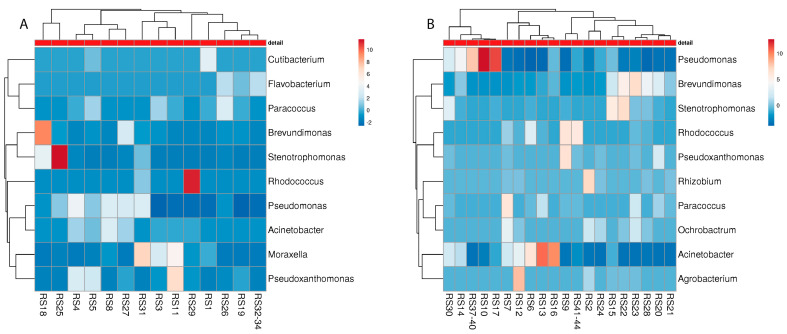
Heatmap of the “best hits” (fractions > 1% of the total quantity) of the bacterial genera identified in the metagenome analysis in different rubber sealants of malodour ((**A**); *n* = 16) and no odour ((**B**), *n* = 26) households. The number of samples shown may differ from the true sample quantity, as individual samples were pooled in the metagenome analysis.

For the rubber sealant, *Pseudomonadaceae* again turned out to be the most common bacterial colonizer (Figure 6). However, other microbial genera were identified, which were not that abundant in the detergent drawer. Inter alia, *Cutibacterium* and *Moraxella* were present in the M machines while *Rhizobium* and *Agrobacterium* were only found in the NM machines. Again, other soil bacteria, such as *Rhodococcus*, could be identified in both the M and NM machines.

**Figure 7:**
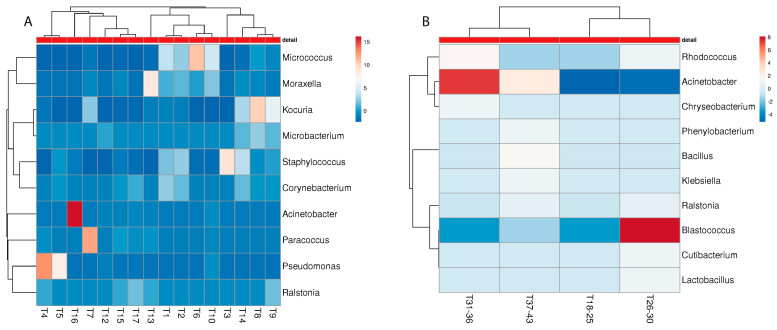
Heatmap of the “best hits” (fractions > 1% of the total quantity) of the bacterial genera identified in the metagenome analysis of different towels of malodour ((**A**); *n* = 16) and no-odour ((**B**), *n* = 26) households. The number of samples shown may differ from the true sample quantity, as individual samples were pooled in the metagenome analysis.

Although towels harboured microbial communities that are different from the sampling sites inside the washing machine, there are some considerable consistencies in terms of malodour relations (Figure 7). Again, different soil bacteria were identified on the NM towels: *Rhodococcus*, *Blastococcus*, and *Phenylobacterium*, whereas on the M towels, the genera *Moraxella*, *Staphylococcus*, *Corynebacterium*, and *Micrococcus* were present, which have previously been associated with malodour [18,26].

### 3.5. Risk Assessment

To understand the possible risk of infection and how adverse microbial effects such as malodour develop, it seems necessary to understand the interplay between microbial communities in the washing machine and on laundered items, since it has already been shown that laundering creates a complex microbial exchange pattern [5]. Interestingly, most of the studies related to the microbiological effects of laundering did not consider the actual microbial burden in vivo and in general, the “natural” bacterial counts on household textiles or in washing machines have only been investigated in a few studies [4,28] (unfortunately, some of the following studies did not provide bacterial counts for a defined surface, so some numbers are only approximated). In 2013, Stapleton et al. detected up to 10^4^ cfu/cm^2^ in the detergent drawer and up to 10^5^ cfu/cm^2^ in the rubber sealant of washing machines [28]. With regard to textiles laundered in a household washing machines, Munk et al. showed in 2001 how the bacterial counts on cotton and polycotton may develop after washing and found 1 x 10^5^ and 1 × 10^4^ cfu/cm^2^ on the day after laundering, respectively [4]. In 2014, Lucassen et al. found a mean total viable count (TVC) of approximately 10^2^ cfu/cm^2^ on hand towels that had been used normally for one week [29]. In addition to the household-related publications, there have been some studies investigating the bacterial burden on textiles in health care facilities [30,31,32]. In this regards, Blaser et al. (1984) found total bacterial counts on objects such as soiled bed sheets and terry towels of 10^4^–10^6^ cfu/cm^2^ [33].

To the best of our knowledge, the present study is the first to provide a quantitative and qualitative comparison of bacterial communities in washing machines and on normally used hand towels that were laundered in these machines. Our results suggest a TVC of aerobic mesophilic bacteria in the washing machine of approximately 10^5^ cfu/cm^2^ while the TVC on normally used items was 10^2^ cfu/cm^2^ after use. These findings are generally consistent with the studies mentioned above and provide proof of a strong bacterial colonization of the detergent drawers and rubber sealants in household washing machines. However, we also showed that the bacterial burden on used and unwashed textiles in standard households can be considered rather low compared to the microbial counts on textiles in clinical settings, confirming the data of Lucassen et al. [29]. We did not analyse the microbial reduction on the used textiles that can be achieved by laundering, since the reduction factors that are typical for domestic laundering procedures are well known from other studies [2,5,34,35,36,37]. However, the cross-contamination by washing machine biofilms must be considered and has not yet been investigated comprehensively, except for a few studies suggesting a considerable input of machine-borne microorganisms [5,29].

When evaluating disinfecting procedures or products, textile test carriers are currently still artificially contaminated with a bacterial count of 10^8^ cfu/cm^2^ according to the normative procedures [38,39]. Given the results of this study, this bacterial count must be considered as being more related to a situation in the health care sector than to the household. A new standard (prEN 17658) explicitly focuses on “chemical textile disinfection for the domestic area” [40] and uses initial counts of >10^6^ cfu/cm^2^ for bacteria and >10^5^ cfu/cm^2^ for yeasts. According to this standard, a reduction of 4 log is required for bacteria and 3 log levels for fungi. Based on the current results, these requirements reflect a consumer-related situation slightly better than the demands given by EN 16616 [38], although it is difficult to define the requirements for antibacterial effects associated with domestic laundering, since the results of the present study do not include situations of higher risks, such as infections. Nevertheless, it must be assumed that used and unwashed textiles may pose a certain risk of infection, since we were able to identify several pathogenic bacterial species by means of a metagenome analysis of the used towels and the washing machine (Table 1). Although the general infection risk may be low, since in most of the cases the actual count of a pathogenic species found on a used textile will certainly be lower than the infectious dose, bacterial numbers may rise over time, especially when the textiles are stored in a damp condition, which is often the case with towels.

In total, thirteen facultative pathogenic strains were identified in this study. Amongst other bacteria associated with humans, staphylococci and *Cutibacterium acnes (C. acnes)* were especially regularly detected on the towel samples. In contrast, the dominant species in the washing machine were water-borne bacteria such as *Pseudomonas aeruginosa (P. aeruginosa)* and *Stenotrophomonas maltophilia (S. maltophilia)*, which have been found in washing machines before [43]. While these data generally support the idea of a low infection risk associated with domestic laundry, our findings clearly show the presence of pathogens on used textiles, which may pose a risk under certain circumstances.

Microbial patterns lead to or protect against laundry malodour. When correlating the occurrence of laundry-related malodour with the quantitative bacterial colonization, no significant differences in the bacterial counts with regards to malodour were found (Figure 4) apart from significantly higher amounts of *Staphylococcus* spp. in the rubber sealant (*p* > 0.05) of the M machines.

While the amount of bacteria does not seem to be a suitable means to explain the development of laundry-associated malodour, the qualitative analysis of the M and NM samples revealed interesting insights. In general, our findings comprise a “typical” bacterial washing machine colonization and confirm the data of Nix et al. (2015) and Jacksch et al. (2020), who showed that *Brevundimonas* sp., *Pseudomonas* sp., *Methylobacterium* sp., *Acinetobacter* sp., and *Rhizobium* sp. were the dominant species in domestic washing machines [7,17]. Nonetheless, these microbial communities have not been related to the formation of malodour yet, except for singular findings, such as linking the presence of *M. osloensis* with a musty textile odour [18,44]. Since, in the present study, *Moraxella* could not be found in all samples from households with malodour experience, it needs to be questioned if one single species may be responsible for the development of laundry malodour.

Recently, Zinn et al. showed that a combination of *Micrococcus luteus (M. luteus)*, *Staphylococcus hominis (S. hominis)*, and *Corynebacterium jeikeium (C. jeikeium)* also led to the formation of malodour [26]. With *Staphylococcus* and *Micrococcus*, two of these genera were detected on malodour towels in the current investigation as well. Gattlen et al. were also able to isolate staphylococci from washing machines using a culture-dependent approach [6]. Furthermore, Madsen et al. were able to show that *S. hominis* was found in 13–25% of living rooms (indoor air) [45]. A similar study by Kooken et al. showed that about two-thirds of the environmental samples from indoor air were *Micrococcus* [46]. Likewise, Callewaert et al. (2015) found *Micrococcus* sp. on worn cotton clothing and suggested that skin-derived staphylococci and *Corynebacteria* are enriched on textiles during washing while *Micrococci* remain abundant [5].

However, the Shannon diversity of the bacterial communities was significantly lower (*p* < 0.05) in NM (exp (H′) = 10.56 ± 9.38) compared to M detergent drawers (exp (H′) = 15.03 ± 7.28). Likewise, significant differences between the M rubber sealants (exp (H′) = 10.57 ± 8.02) and NM rubber sealants (exp (H′) = 5.03 ± 4.09) were observed (Table 2).

In contrast, there were no significant differences between NM (exp (H′) = 2.75 ± 1.24) and M towels (exp (H′) = 7.25 ± 5.56). These results might suggest a protective function of high microbial diversity with regards to malodour and support the hypothesis of a multifactorial origin of laundry malodour rather than a single species being responsible for odour development.

**Figure 8:**
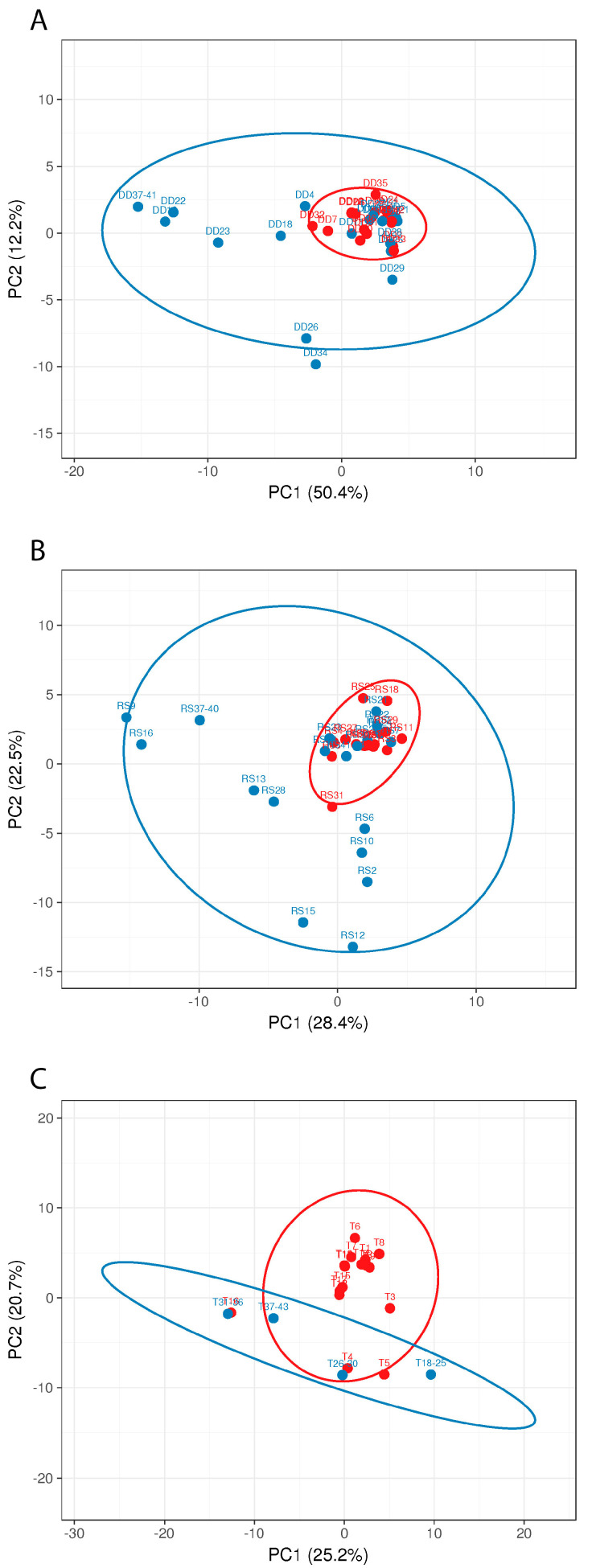
Principle component analysis (PCA) of the different sampling sites ((**A**): detergent drawer; (**B**): rubber sealant; (**C**): towel M and (red, *n* = 16) and NM (blue, *n* = 26) households. The number of samples shown may differ from the true sample quantity, as individual samples were pooled in the metagenome analysis.

To further investigate the bacterial colonization patterns in the M and NM samples, principal component analysis (PCA) was performed for all sampling sites (Figure 8).

The PCA clearly shows that the M samples from all three sampling sites form clusters, which, for the detergent drawer and the rubber sealant, lie within the NM samples’ distribution, suggesting a distinct yet not completely different colonization pattern for malodorous machines. In contrast, the M samples from the investigated towels differentiate more clearly from the NM samples. The species that were only present in the NM machines may exert their protective properties by replacing malodour-producing species or further metabolizing malodorous substances. Although PCA suggests a role for microbial colonizers of the washing machine and microorganisms present on the used textile for malodour formation, it cannot be excluded that microbial contaminants that are introduced after laundering (e.g., during drying) might influence malodour formation as well.

**Figure 9:**
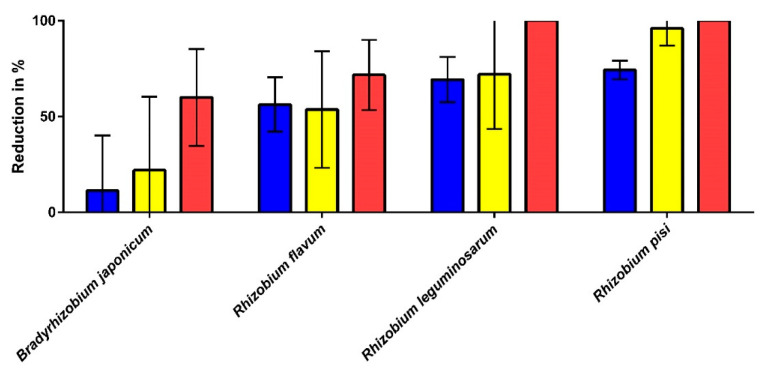
Malodour reduction by *Bradyrhizobium* and *Rhizobium* strains for general malodour (blue), cheesy odour (yellow), and pungent odour (red) using the malodour model described in [26] (*n* = 5). All reductions shown are in comparison to the positive control (very high proportion of the three odour attributes). The samples were evaluated by a trained sniffer panel. The error bars have been shortened to the bottom for reasons of clarity.

To prove the idea of malodour-protecting bacteria, we used a model to generate laundry-associated malodour in vitro that was published by Zinn et al. in 2021 [26]. In this study, it was shown that, amongst other substances, dimethyl disulfide, dimethyl trisulfide, and indole may be particularly accountable for the wet-fabric-like malodour. Apart from being volatile malodorous substances, Weisskopf and co-workers recently showed that dimethyl disulfide and dimethyl trisulfide inhibit the growth of soil bacteria and promote the growth of *Pseudomonas* sp. [47], which is in line with the identified microbial community patterns of the M and NM rinsing chambers in the present study. As *Rhizobium* was one of the soil bacteria found in the present analysis and was frequently mentioned in the literature, its impact on the malodour model according to Zinn et al. [26] was investigated. For this purpose, we used *Rhizobium flavum (R. flavum)* and *R. leguminosarum* identified in the metagenome analysis of the current study and other species of soil bacteria (Figure 9).

The evaluation of the malodour model shows that the two *Rhizobium* species found in the washing machines (*R. flavum* and *R. leguminosarum*) lead to a reduction of >50% of the malodour in general and the cheesy and pungent odours. Furthermore, *Rhizobium pisi*, was found to show an equally good reduction against the three odour attributes. *Bradyrhizobium japonicum*, which was tested as a control, only showed minor reductions against general malodour (−11.3%) and the cheesy (−22%) and pungent odours (−60%).

Thus, together with their predominant presence in NM machines, these findings strongly suggest that *Rhizobium* spp. have a positive impact on laundry-related malodour. More research is needed, however, to reveal the exact interrelationship of malodorous and (putatively) protective bacterial species, since although our data hint at *Rhizobium* spp. being a protective means with regards to malodour, we did not find Rhizobia in any of the NM samples, so the potential role of other bacteria still has to be elucidated. Nonetheless, it seems obvious from our observations that the question of whether malodour develops in a laundry-related environment must be considered as a complex interplay between numerous bacterial groups rather than an effect caused by a distinct species. Moreover, there may be other microorganisms, which were not focused on here, such as fungi, contributing to this phenomenon as well.

## 4. Conclusions

During the whole wash-and-use cycle, textiles interact with numerous microorganisms in a complex way [2]. While microbial cells are introduced onto the textile surfaces by wearing, storage, and even by the washing machine itself [29], microbial reduction takes place, e.g., when laundering or drying the garments, resulting in an ongoing exchange of the microbial community in the textile [5]. To understand the role of the textile and washing machine microbiota in the putative infection risk and other adverse effects, such as malodour, microbiological investigation of the whole wash-and-use-cycle is needed. The present study provides a comprehensive quantitative and qualitative comparison of the bacterial communities in washing machines and on normally used hand towels that were laundered in these machines, thus enabling a better risk assessment of laundry-associated infections and suggesting possible mechanisms leading to other problems related to laundry-associated microorganisms, such as malodour formation. Although the explanations given by this study are not exhaustive, we identified soil bacteria as a group of microbial colonizers of washing machines that might act as a kind of protective factor against laundry malodour and thus should be investigated further. Hence, in the future, changing the laundry-associated microbial communities might be a promising way to handle microbial risks and unwanted effects in a sustainable way.

## Figures and Tables

**Table 1:** The 50 most common species in detergent drawers and on rubber sealants and various towels presented as the relative frequency in % of the total microbial composition (nd = not defined in [40,41]).

Species	Risk Group (According to [41,42])	Detergent Drawer (in %)	Rubber Sealant (in %)	Hand Towel (in %)	Body Towel (in %)	Kitchen Cloth (in %)
*Actinomycetospora succinea*	1	0.21	0.02	0.02	0.00	0.00
*Agrobacterium tumefaciens*	1	0.51	0.36	0.02	0.00	0.00
*Aspergillus glaucus*	1	0.00	0.00	0.73	0.00	0.00
*Aureimonas altamirensis*	1	0.56	0.03	0.01	0.00	0.00
*Bacillus subtilis*	1	0.01	0.00	2.89	0.00	0.00
*Brevundimonas bullata*	1	0.22	0.27	0.00	0.00	0.00
*Brevundimonas diminuta*	1	0.17	0.30	0.01	0.00	0.00
*Brevundimonas sp DS20*	1	0.13	0.23	0.00	0.00	0.00
*Brevundimonas sp SH203*	1	0.35	0.31	0.01	0.00	0.01
*Flavobacterium lindanitolerans*	1	0.05	0.26	0.00	0.00	0.00
*Homo sapiens*	1	0.03	0.24	0.24	2.17	0.15
*Kocuria rhizophila*	1	0.01	0.07	1.60	0.07	0.01
*Methylorubrum extorquens*	1	0.23	0.00	0.01	0.00	0.00
*Micrococcus luteus*	1	0.22	0.16	2.54	0.02	0.01
*Mycolicibacterium tusciae*	1	0.19	0.05	0.01	0.00	0.00
*Pseudomonas fluorescens*	1	0.20	0.15	0.14	0.03	0.02
*Pseudomonas fragi*	1	0.01	0.12	0.01	6.57	4.49
*Pseudomonas oleovorans*	1	0.04	0.27	0.00	0.00	0.00
*Pseudomonas veronii*	1	0.29	0.05	0.00	0.00	0.00
*Pseudoxanthomonas spadix*	1	0.02	0.58	0.00	0.00	0.00
*Pseudoxanthomonas suwonensis*	1	0.11	0.39	0.00	0.00	0.00
*Rhizobiales bacterium*	1	0.16	0.21	0.01	0.00	0.01
*Rhizobium sp ACO-34A*	1	0.00	0.34	0.00	0.00	0.00
*Rhodococcus erythropolis*	1	0.04	0.10	0.18	0.17	0.20
*Skermanella aerolata*	1	0.22	0.10	0.00	0.00	0.00
*Staphylococcus warneri*	1	0.00	0.00	0.66	0.02	0.00
*Stenotrophomonas rhizophila*	1	0.06	0.20	0.00	0.00	0.07
*Xanthobacter autotrophicus*	1	0.37	0.08	0.00	0.00	0.00
*Xanthobacter tagetidis*	1	0.04	0.26	0.00	0.00	0.00
*Acinetobacter johnsonii*	2	0.17	0.47	0.16	0.00	3.00
*Acinetobacter ursingii*	2	0.26	0.32	0.03	0.00	0.55
*Brevibacterium casei*	2	0.57	0.03	0.11	0.01	0.00
*Cutibacterium acnes*	2	0.02	0.15	0.54	12.26	0.21
*Moraxella osloensis*	2	0.42	2.75	3.26	0.05	0.60
*Paracoccus yeei*	2	0.11	0.37	0.74	0.00	0.03
*Pseudomonas aeruginosa*	2	1.04	0.35	2.84	0.07	0.05
*Pseudomonas alcaligenes*	2	0.20	0.07	0.00	0.00	0.00
*Pseudomonas stutzeri*	2	0.64	0.50	0.08	0.00	0.01
*Roseomonas gilardii*	2	0.21	0.03	0.05	0.00	0.00
*Staphylococcus aureus*	2	0.00	0.02	1.40	0.16	0.04
*Staphylococcus epidermidis*	2	0.02	0.01	0.47	0.44	0.02
*Stenotrophomonas maltophilia*	2	0.70	1.54	0.04	0.01	0.02
*Aquabacterium sp SJQ9*	(nd)	0.64	0.03	0.00	0.00	0.00
*Blastococcus sp CCUG 61487*	(nd)	0.02	0.20	0.05	0.00	0.00
*Janibacter indicus*	(nd)	0.14	0.23	0.04	0.00	0.00
*Micavibrio aeruginosavorus*	(nd)	0.01	0.63	0.00	0.00	0.00
*Paracoccus salipaludis*	(nd)	0.00	0.02	0.52	0.00	0.00
*Phenylobacterium sp Root700*	(nd)	0.36	0.19	0.25	0.00	0.00
*Rahnella inusitata*	(nd)	0.00	0.00	0.01	3.03	1.28

**Table 2:** Shannon diversities of the bacterial genera identified in the metagenome analysis in different towels of malodour (M, *n* = 16) and no-odour (NM, *n* = 26) households.

Sample	Shannon Diversity	Standard Deviation
M detergent drawer	15.03	7.28
NM detergent drawer	10.56	9.38
M rubber sealant	10.57	8.02
NM rubber sealant	5.03	4.09
M towel	7.25	5.56
NM towel	2.75	1.24

## Data Availability

Not applicable.

## Supplementary Materials

The following supporting information can be downloaded at: https://www.mdpi.com/article/10.3390/microorganisms10071465/s1, Table S1: Laundry hygiene questionnaire. Table S2: Information on household and towel use (questionnaire).
